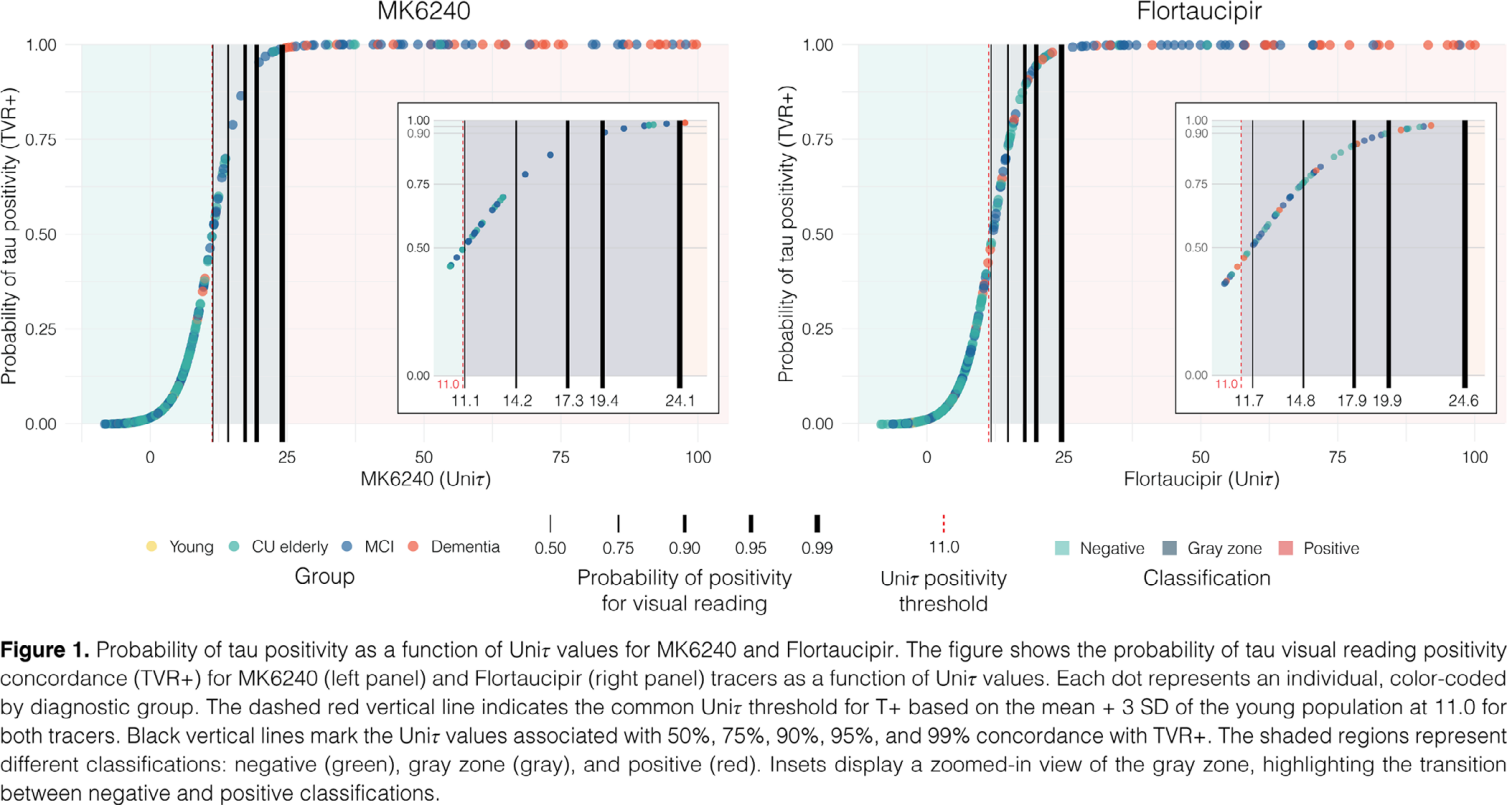# Defining tau PET positivity grey zones for MK6240 and Flortaucipir quantification

**DOI:** 10.1002/alz70862_109951

**Published:** 2025-12-23

**Authors:** Guilherme Povala, Bruna Bellaver, Guilherme Bauer‐Negrini, Emma Ruppert, Marina Scop Medeiros, Livia Amaral, Firoza Z Lussier, Pamela C.L. Ferreira, Carolina Soares, Dana L Tudorascu, Quentin Finn, Joseph C. Masdeu, Hwamee Oh, Juan Fortea, David N. soleimani‐Meigooni, Val J Lowe, Brian A. Gordon, Belen Pascual, Pedro Rosa‐Neto, Suzanne L. Baker, Tharick A Pascoal

**Affiliations:** ^1^ University of Pittsburgh, Pittsburgh, PA USA; ^2^ Houston Methodist Research Institute, Houston, TX USA; ^3^ Brown University, Providence, RI USA; ^4^ Sant Pau Memory Unit, Hospital de la Santa Creu i Sant Pau, Biomedical Research Institute Sant Pau, Barcelona Spain; ^5^ University of California, San Francisco, San Francisco, CA USA; ^6^ Mayo Clinic, Rochester, MN USA; ^7^ Washington University in St. Louis, St. Louis, MO USA; ^8^ McGill University, Montreal, QC Canada; ^9^ Lawrence Berkeley National Laboratory, Berkeley, CA USA

## Abstract

**Background:**

Tau PET measures are inherently continuous and applying dichotomized thresholds introduces conceptual and analytical idiosyncrasies. Understanding the limitations in the transition from tau‐negative to tau‐positive classifications is crucial for the effective use of these thresholds. This study aims to determine the confidence levels of tau PET thresholds of abnormality for different tau PET tracers by characterizing their "gray zone" using the universal tau PET scale (Uniτ, www.unitau.app).

**Method:**

We evaluated 485 individuals across the aging and AD spectrum from the HEAD study, with head‐to‐head scans for MK6240 and Flortaucipir. Uniτ estimates were derived from the Meta‐Temporal ROI. Tau positivity (T+) was defined as Uniτ values exceeding the mean plus 3 SD of individuals younger than 28 years (*n* = 24). Two physicians independently performed a visual assessment of tau positivity for each tracer, with agreement indicating clear tau pathology (TVR+). Logistic regression was used to estimate the probability of TVR+ across Uniτ values for each tracer (*n* = 189, CU elderly Aβ‐ and CI Aβ+). Individuals were classified as negative, positive, or within a "gray zone" between the most liberal T+ threshold and varying TVR+ probability thresholds (50%, 75%, 90%, 95%, and 99%).

**Result:**

The Uniτ gray zone, defined as a function of TVR+ probabilities, demonstrated consistency between the two tracers, with differences observed only in the decimal range. The most liberal Uniτ threshold for T+ was 11.0 for both MK6240 and Flortaucipir, closely matching the 50% TVR+ probability threshold (11.1 for MK6240 and 11.7 for Flortaucipir). Higher TVR+ probabilities corresponded to increased Uniτ values with MK6240 and Flortaucipir showing near‐identical values between the tracers: 14.2 and 14.8 for 75%, 17.3 and 17.9 for 90%, 19.4 and 19.9 for 95%, and 24.1 and 24.6 for 99%, respectively (Figure 1). In total, 26 participants fell within the gray zone for MK6240, compared to 47 participants for Flortaucipir.

**Conclusion:**

These findings highlight the potential of Uniτ to provide a standardized approach for assessing tau positivity across tracers. By combining quantitative Uniτ measures with visual assessments, we enhance the understanding of tau positivity certainty, particularly in the transition between negative and positive classifications.